# Altered resting-state electroencephalography microstate characteristics in young male smokers

**DOI:** 10.3389/fpsyt.2022.1008007

**Published:** 2022-10-04

**Authors:** Xiaojian Li, Fang Dong, Yunmiao Zhang, Juan Wang, Zhengxi Wang, Yaning Sun, Ming Zhang, Ting Xue, Yan Ren, Xiaoqi Lv, Kai Yuan, Dahua Yu

**Affiliations:** ^1^Inner Mongolia Key Laboratory of Pattern Recognition and Intelligent Image Processing, School of Information Engineering, Inner Mongolia University of Science and Technology, Baotou, China; ^2^College of Information Engineering, Inner Mongolia University of Technology, Hohhot, China; ^3^School of Life Sciences and Technology, Xidian University, Xi’an, Shaanxi, China

**Keywords:** young smokers, microstate, resting state, electroencephalography (EEG), nicotine addiction

## Abstract

The development of nicotine addiction was associated with the abnormalities of intrinsic functional networks during the resting state in young adult smokers. As a whole-brain imaging approach, EEG microstate analysis treated multichannel EEG recordings as a series of quasi-steady microscopic states which were related to the resting-state networks (RSNs) found by fMRI. The aim of this study was to examine whether the resting-state EEG microstate analysis may provide novel insights into the abnormal temporal properties of intrinsic brain activities in young smokers. We used 64-channel resting-state EEG datasets to investigate alterations in microstate characteristics between twenty-five young smokers and 25 age- and gender-matched non-smoking controls. Four classic EEG microstates (microstate A, B, C, and D) were obtained, and the four temporal parameters of each microstate were extracted, i.e., duration, occurrence, coverage, and transition probabilities. Compared with non-smoking controls, young smokers showed decreased occurrence of microstate C and increased duration of microstate D. Furthermore, both the duration and coverage of microstate D were significantly negatively correlated with Fagerstrom Test of Nicotine Dependence (FTND) in young smoker group. The complex changes in the microstate time-domain parameters might correspond to the abnormalities of RSNs in analyses of FC measured with fMRI in the previous studies and indicate the altered specific brain functions in young smokers. Microstate D could be potentially represented as a selective biomarker for predicting the dependence degree of adolescent smokers on cigarettes. These results suggested that EEG microstate analysis might detect the deviant functions of large-scale cortical activities in young smokers and provide a new perspective for the study of brain networks of adolescent smokers.

## Introduction

Nicotine is an important reason for the high incidence of mental and physical illness, which has killed more than 5–6 million people per year worldwide and increased the overall burden of disease (1–3). As a result, the negative effects of smoking go far beyond individual and population health. According to the report on the health hazards of smoking in 2020 issued by the National Health Commission of China, the smoking rate of people over 15 years old in China is 26.6% in 2018, of which the smoking rate of men is 50.5%.^1^ Despite the global decline in smoking, the serial national cross-sectional surveys in China from 2003 to 2013 report that the smoking rate among adolescent smokers, aged 15–24, increase from 8.3% in 2003 to 12.5% in 2013 (4). When exposed to nicotine in the early stages of development, the neurotoxic effects of nicotine are more serious (5). People who start smoking at an earlier age are more likely to be addicted to nicotine and become lifelong smokers (6). Previous studies have shown that neurophysiology of the brain in adolescent smokers undergoes a series of persisting and important developments and changes (7–9). Exposure to nicotine during adolescence induces continuous changes in neural connections (10), as well as some changes in emotional and cognitive functions (11–14). Even though smokers realize the negative effects of smoking and express a strong desire to quit, most attempts to quit smoking end in relapse (15).

Previous studies suggested that deviant neural brain activities in young smoker are not confined to any particular brain region (16–18). The development of nicotine addiction was particularly associated with the resting-state functional connectivity (FC) networks (19, 20), including salience network (SN), default mode network (DMN), and central executive network (CEN) (21, 22). Studies have shown the abnormalities of the resting-state FC in the insula and putamen and significant correlations between the resting-state FC strength and clinical information (pack-years, craving and FTND) in young smokers (19, 20). The resting-state networks (RSNs) produced by the signal blood oxygen level-dependence (BOLD) fluctuations of spontaneous functional magnetic resonance imaging (fMRI) reflected a constant inner state of exploration, in which the brain produces predictions about possible network configuration, thus influencing cognitive processing and perception (23). In a resting-state fMRI study, the abnormalities of RSNs were found in analyses of FC in adolescent smokers, which were reflected in the enhancement of FC in the DMN, CEN and SN networks and the significant correlation between the increase of FC in the networks and smoking behavior (17). A resting-state electroencephalography (EEG) study found the decrease in global network efficiency assessed by the minimum spanning tree in young male smokers (16). Although the aberrant activities of the functional networks in brain had been found in young smokers, more studies on the aberrant organization and function of large-scale neural networks were needed to further explore the neural mechanism of smoking addiction in adolescent.

Electrical activity in the central areas of human brain can be obtained at a high temporal resolution via electroencephalography (EEG), which records the electric potential distributions within the brain through the electrodes on the surface of scalp. Among all the quantitative EEG techniques, one ordinary approach regards the EEG signals as a dynamic system which can be described according to its dynamics and state (24). EEG microstate analysis is one of the whole brain imaging approaches that can characterize the spatial organization and temporal dynamics of large-scale cortical activities with high temporal resolution (25). It considers multichannel EEG records to be a series of quasi-steady microscopic states, each of which is characterized by the unique topographic topology of the entire channel (24). Consequently, EEG microstate analysis could not only analyze the abnormal dynamics of the whole brain networks, but also evaluate the function of large-scale brain networks associated with several neuropsychiatric disorders.

The EEG microstates revealed that the morphological changes of electric field in the brain were nonlinear and discontinuous. A given head-surface topography of electric field in the brain tended to stay a quasi-stable state for a period of approximately 80–120 ms before transforming into another different topography (26). These quasi-stable topographic periods were called microstates, which reflected the basic steps information processing of brain in spontaneous and event-related researches (27). In general, the scalp topographies were clustered into four classes of maps, labeled as microstates A, B, C, and D, that could explain about 80% of the variance of electric potential changes in EEG (28). The four topographies were very similar in different mental states, age groups, or in health and disease, but their inherent characteristics (e.g., duration, occurrence rate, and time coverage) were modulated by neuropsychiatric disorders, personality types and cognitive manipulations (29). Present studies demonstrated that EEG microstates had significant changes in some central neuropsychiatric diseases and EEG microstate analysis might be an effective method for perceiving objective indicators, identifying the severity of disease and formulating treatment plans (24). Moreover, some studies with simultaneous EEG and fMRI found that the four main EEG microstates seemed to express the neurophysiological correlate with four RSNs assessed by fMRI (30). Therefore, the existence of characteristics in specific microstates might be regarded as quantifiable state markers of different neurological and neuropsychiatric disorders (31).

Although the spontaneous EEG microstate analysis has been used to evaluate the whole-brain coordinating changes associated with brain maturation, so far, knowledge of abnormal microstate in young adult smokers is limited. Thus, in this work, the alterations of EEG microstates were detected between young adult smokers with healthy non-smoking controls, aiming to explore the neurophysiological mechanism of young smokers. These quantifiable features in specific microstates could be considered as the useful state markers in the diagnosis and treatment for nicotine addiction in young adult smokers. With the alterations in the neural activity of young adult smokers and non-smoking controls, we hypothesize that the microstates changed significantly in young adult smokers during the resting state. Additionally, the association between microstate characteristics and clinical scale scores was investigated to find the relation between dynamic brain activities and smoking status.

## Materials and methods

### Ethics statement

This study was approved by the Medical Ethics Committee of the First Affiliated Hospital of Baotou Medical College of Inner Mongolia University of Science and Technology, and conformed to the Declaration of Helsinki. All procedures were performed with the full understanding the purpose of the study and written informed consent of the subjects and their legal guardian.

### Participants

Twenty-five young male smokers (mean age: 19.58 ± 1.99 years) and an equal number of young male non-smoking controls (mean age: 19.05 ± 1.72 years) were recruited from Inner Mongolia University of Science and Technology. The inclusion criteria for smokers were as follows: (1) meet the current DSM-V criteria for nicotine dependence; (2) nicotine dependence test assessed by Fagerström Test for Nicotine Dependence (FTND) (32) ≥3 points; (3) smoked 10 or more cigarettes a day for the past 6 months; (4) quit smoking for no more than 3 months; (5) expired air carbon monoxide (CO) >6 ppm. Non-smoking controls: (1) smoked less than three cigarettes in their lifetime; (2) their parents and roommates were not smokers (to avoid the effects of second-hand smoke). None of the participants found any physical, neuropsychiatric or extracranial lesions caused by alcohol or drug abuse. Demographic and smoking characteristics of the participants were presented in Table 1.

**TABLE 1:** Demographic and smoking characteristics of the participants.

Clinical details	Smokers (*n* = 25)	Non-smokers (*n* = 25)	*P*-value
Age (years)	20.40 ± 1.26	20.16 ± 1.14	0.5
Age range (years)	18∼23	18∼23	–
Education (years)	14.04 ± 0.61	14.24 ± 0.66	0.3
Cigarettes per day (CPD)	14.25 ± 4.59	–	–
Age of smoking initiation	15.33 ± 2.78	–	–
Duration of smoking	4.21 ± 2.21	–	–
Pack-Years	2.95 ± 2.73	–	–
FTND score	4.56 ± 1.50	–	–
Profitable hand	Right handedness	Right handedness	–

Values are expressed as means ± standard deviations. Pack-years: Duration of smoking × CPD/20. FTND, Fagerström Test for Nicotine Dependence. All variables were compared between groups with the independent samples *t*-test.

*P* < 0.05.

### Electroencephalography recording

The experimental environment in which data was collected could greatly reduce the influence of external factors, so as to obtain more accurate the resting-state EEG data. The data collection was conducted in a comfortable, quiet, soundproof and dimly lit EEG laboratory at room temperature. The EEG signals were recorded with two BrainAmp MR plus amplifiers (Brain Products GmbH. Munich. Germany) from 64 scalp sites (positioned according to the 10–20 International System) with Ag/AgCl electrodes. Two active electrodes were placed on the outer canthus of right eye and above the left eye for recording the vertical electrooculogram (EOG). All EEG signals were digitalized with a sample rate of 1,000 Hz with a frequency band from 0.1 to 250 Hz, and electrode impedances were reduced to less than 10 kΩ. The resting-state data were collected for 10 min with the participant’s eyes closed.

### Data preprocessing

The offline data preprocessing was performed in the MATLAB R2016a platform (The Mathworks Inc., Natick, MA, United States) using the EEGLAB toolbox (33). The continuous signals from 2 to 7 min in the middle of the resting-state data for each participant were selected. The following steps were applied: (1) the EEG data were re-referenced to the average signal of all the electrodes; (2) the signals were band-pass filtered from 1 to 20 Hz; (3) The sampling rate was dropped to 250 Hz; (4) portions of the data contaminated by eye movements, blinks, electromyography, electrocardiography or any non-physiological artifacts were corrected by independent component analysis (ICA) (34); (5) the signals were segmented into 2 s epochs. (6) Then, the strong muscle artifacts with amplitude values exceeding 80 μv at any electrode were manually rejected. Participants with at least 100 epochs were used for microstate analysis.

### Microstate analysis

Microstate analysis was performed using the microstate EEGLAB toolbox (35) in MATLAB to detect and compute the characterization of EEG microstates. First, the global field power (GFP) which reflected the variation degree in the potential across electrodes at a certain moment was extracted, and all topographic maps at the peak of GFP were obtained. The minimum peak distance was 10 ms. The number of GFP peaks entering the segmentation area for each sample was 1,000. The GFP peaks exceeding 2 times the standard deviation of all GFP peaks were eliminated. For microstate segmentation, the selected GFP peaks for all subjects were aggregated into a file to maximize the similarity between the microstate prototypes they were assigned to and the EEG samples, using the modified K-means clustering algorithm in this study because it ignored the polarity of the EEG (27). The number of microstate categories was defined as four, which was the optimal number of categories consistent with previous studies (24, 27). During microstate segmentation, the number of repetitions was 50 and the maximum number of iterations was increased to 1,000 to find a trade-off between precision of the segmentation and computation time. After the microstate prototypes were obtained, they were fitted back to all of the continuous EEG signals by means of spatial correlation between the EEG data and the microstate prototype maps and the resultant highest spatial correlation was used to determine which microstate the present EEG data belonged to. Thus, a continuous sequence of microstates on a time scale was obtained. Since the EEG signals in the resting state were noisy, this noise could contribute to the generation of short microstate fragments after clustering or fitting. To address this, we rejected microstate segments less than 30 ms. In this method, the labels of time frames in small microstate fragments were assigned to the next most likely microstate category, which was measured by global map dissimilarity (35). Finally, in order to compare the differences in EEG microstates between young adult smokers and non-smoking controls, the characteristic microstate parameters were calculated: (1) Duration: the average duration time of a given microstate category; (2) Occurrence: the average number of times per second that a microstate was dominant; (3) Coverage: the fraction of time covered by a given microstate category; (4) Transition probabilities: the probabilities of transition from a certain microstate class to another classes.

### Statistical analysis

The statistical analysis was carried out in SPSS 20.0 software (SPSS Statistics, IBM, Armonk, NY, United States). Independent sample *t*-test was used to compare the significant differences in demographic data (age, education), smoking characteristics (packet year, FTND, CPD, age of smoking initiation and duration of smoking), and microstate time-domain parameters between smokers and non-smoking controls. Eventually, Spearman correlation coefficients were calculated to evaluate the correlation between microstate features and smoking characteristics.

## Results

### Demographic information

Demographic analysis showed no significant differences in the distributions of age and education between the two groups. According to the self-reports of smoking characteristics, the number of cigarettes per day was 14.25 ± 4.59 in young smokers, the initial smoking age was 15.33 ± 2.78 years, the smoking duration was 4.21 ± 2.21 years, the mean FTND score was 4.75 ± 1.48 and the pack-years was 2.95 ± 2.73 (Table 1).

### Electroencephalography microstate results

**FIGURE 1:**
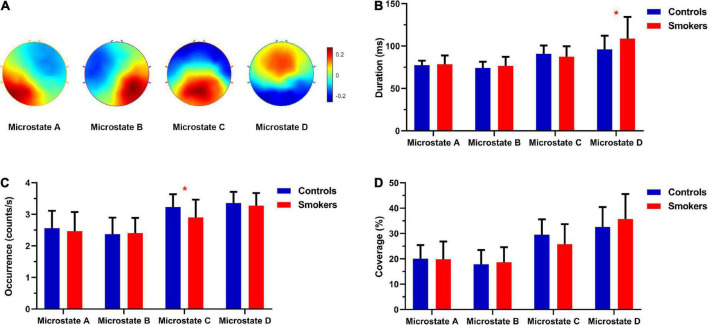
**(A)** The topographies of the four microstate classes that were calculated based the aggregated dataset from all participants. **(B)** The differences in the microstate duration between young smokers and non-smoking controls. **(C)** The differences in the microstate occurrence between young smokers and non-smoking controls. **(D)** The differences in the microstate coverage between young smokers and non-smoking controls. *Indicated significant difference (*p* < 0.05).

Figure 1A showed the topographies of the four microstate classes for the smokers and non-smoking controls, which highly resemble those obtained in previous researches (36). Figures 1B–D showed the differences in the microstate parameters including the duration, occurrence and coverage between two groups. We found that the occurrence of microstate C was decreased significantly in young smokers compared with non-smoking controls (*t* = 2.357, *df* = 50, *p* = 0.023). The duration of microstate D was increased significantly in the smoking group compared with the control group (*t* = 2.088, *df* = 50, *p* = 0.042). Additionally, compared with the non-smoking controls, the coverage of microstate D in the smoking group was increased, but not significant (*t* = 1.9, *df* = 50, *p* = 0.064). No significant differences were revealed in the transition probabilities among microstates.

### Correlation results

**FIGURE 2:**
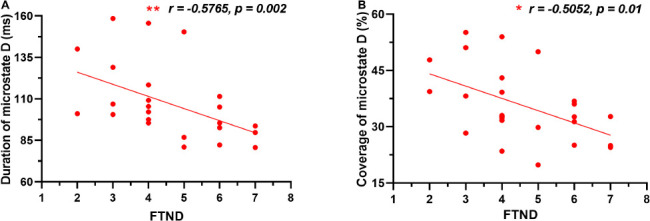
Correlations between microstate features and smoking characteristics of young smokers. **(A)** The duration of microstate D was significantly negatively correlated with FTND in young smokers. **(B)** The coverage of microstate D in young smokers was significantly negatively correlated with FTND.

Figure 2A demonstrated that the duration of microstate D was significantly negatively correlated with FTND in young smokers (*r* = −0.5765, *p* = 0.002). While in Figure 2B, the coverage of microstate D in young smokers was significantly negatively correlated with FTND (*r* = −0.5052, *p* = 0.01).

## Discussion

In the absence of stimulus-evoked tasks, the brain showed organized and spontaneous fluctuations of neural activity (37). Recent findings through nicotine addiction suggested that the abnormal intrinsic brain functional connectivity may contribute to the perception of cravings and predict the severity of smoking urges induced by abstinence (17, 38). To further understand aberrant activity within the brains of adolescent smokers, microstate analysis was employed to assess the large-scale brain network function while simultaneously considering signals from all cortical regions. The present studies found the significant differences in the temporal properties of the resting-state EEG microstate categories between young smokers and non-smoking controls. In young smokers, the occurrence of microstate C decreased significantly, and the duration of microstate D increased significantly. Furthermore, we found that both duration and coverage of microstate D were significantly negatively associated with FTND in young smokers’ group.

Microstate analysis has been the goal of several studies, but the functional significance of EEG microstate categories has not been fully established (39–41). Previous studies with simultaneous EEG and fMRI found that the four main EEG microstate categories seemed to correlate significantly with RSNs assessed by fMRI (28, 30). Microstate class A was associated with changes in negative BOLD activation in bilateral superior temporal and middle temporal parietal cortex, reflecting auditory or sensorimotor networks (42). Microstate class B was reported to reflect the visual resting-state networks by way of changes of BOLD activation in striate and extra-striate cortex, while microstate class C was related to activity of cognitive control network and primarily reflected the SN in bilateral insular and anterior cingulate cortex (25). Previous study demonstrated that microstate class D was associated with attentional network involved to signals in right lateral dorsal and ventral regions of frontal and parietal lobe (25).

Microstate class C represented activities in the anterior cingulate cortex (ACC) and the posterior cingulate cortex (PCC), two important hubs of the DMN, whose areas overlap with the SN and were involved in subjective interoceptive-autonomic processing (43). The previous study had reported that the occurrence and duration of microstate C were decreased in serial subtraction task compared with wakeful rest, which suggested that microstate C was associated with the task-negative DMN (41). Our previous findings in analyses of the abnormalities of RSNs showed that young smokers had greater FC within the DMN, CEN and SN (17). In the study by Milz et al. (26), the head surface topography of different microstates was induced mainly by the intensity and spatial distribution of activity in alpha band within the cerebral cortex, and the microstate C was characterized by greater alpha activity in large areas of the cortex. A positive correlation between the contribution of microstate C and vigilance had been observed, which illustrated the role of microstate C in cognitive control processes (44). Loss of vigilance was accompanied by the power shift of alpha band from occipital to frontal regions, and increased the power in delta and theta band (45). In a previous study, microstate C was associated with a idling state during relaxation with eyes closed, and the occurrence of microstate C indicated the disruption of illusionary motion, whereas their occurrence decreased during construction of new perception (46). Our previous study observed a significant positive correlation between the power in alpha band and NoGo errors in inhibition control task for non-smoking controls compared with young smokers (18). It might be speculated that the reduction in microstate C was related to cognitive-emotional assessments by environmental stimuli.

Some studies suggested that all the microstate D parameters could be modulated by processes containing reality testing (39, 47). A meta-analysis in schizophrenia showed patients with shortened duration and reduced coverage of (47). Microstate D also exhibited shortened duration and reduced coverage during deep hypnosis (48), whereas microstate D had prolonged duration and reduced occurrence during non-REM sleep (49). In addition, the duration of microstate D during hallucinations was shortened (50), whereas the duration was increased during follow-up in patients who responded well to antipsychotic medication (51). The increased occurrence and duration of microstate D were observed in serial subtraction task compared with wakeful rest (41). These results indicated that microstate D was related with the frontoparietal attention network (30). However, other authors reported the increased occurrence and duration of microstate D during wakeful rest compared with cognitive tasks, which showed that microstate D represented focus switching and reflex in attention (52). Our results indicated that young smokers showed higher duration in microstate D. This might be consistent with the results from RSN studies assessed by fMRI (17), which reported that the FCs of the dorsolateral prefrontal lobe and the parietal region in the resting brain network were enhanced in the adolescent smokers. Our correlation analysis indicated that degree of nicotine dependence in young smokers was associated to the duration decrease of microstate D as well as the occurrence decrease of class D. Therefore, microstate D could be a selective biomarker that potentially predicted the later degree of nicotine dependence in young smokers.

There were several limitations in the current study. Firstly, the sample size was relatively small. The larger studies should be investigated to explore microstates as classifiers for smoking characteristics. Furthermore, we did not find any correlation between microstate features and specific cognitive functions. This could be due to lower sample size, or the microstate analysis steps to extract values based on the global map. Future studies should continue to investigate the functional significance of EEG microstates in distinct cognitive processes and incorporate larger samples.

## Conclusion

In the current study, we found that young smokers had significantly decreased occurrence of microstate C and increased duration of microstate D compared to non-smoking controls. The changes might correspond to the abnormalities of RSNs in analyses of FC measured with fMRI in young smokers in the previous studies. Furthermore, both duration and coverage of microstate D were significantly negatively associated with FTND in young smoker group. Our study provides a new perspective on the effects of smoking on adolescent brain networks.

## Data availability statement

The original contributions presented in this study are included in the article/supplementary material, further inquiries can be directed to the corresponding authors.

## Ethics statement

The studies involving human participants were reviewed and approved by Medical Ethics Committee of the First Affiliated Hospital of Baotou Medical College of Inner Mongolia University of Science and Technology. The patients/participants provided their written informed consent to participate in this study. Written informed consent was obtained from the individual(s) for the publication of any potentially identifiable images or data included in this article.

## Author contributions

DY, KY, and XLv designed the experiments. YZ, ZW, and YS performed the experiments. XLi and FD analyzed the data and wrote the article. JW, MZ, TX, YR, KY, and DY provided the critical revision of the article. All authors critically reviewed the content and approved the final version for publication.

## Footnotes

1
http://www.nhc.gov.cn/
